# MET-values of standardised activities in relation to body fat: studies in pregnant and non-pregnant women

**DOI:** 10.1186/s12986-018-0281-z

**Published:** 2018-06-20

**Authors:** Elisabet Forsum, Birgitta Janerot-Sjöberg, Marie Löf

**Affiliations:** 1 Department of Clinical and Experimental Medicine, Linköping University, 581 85 Linköping, SE Sweden; 2 Division of Cardiovascular Medicine, Department of Medical and Health Science, Linköping University, Linköping, Sweden; 3 Department of Clinical Science, Intervention and Technology, Karolinska Institute, Stockholm, Sweden; 4 Department of Clinical Physiology, Karolinska University Hospital, Stockholm, Sweden; 5 Department of Biosciences and Nutrition, Karolinska Institute, Huddinge, Sweden; 6 Department of Medical and Health Sciences, Linköping University, Linköping, Sweden

**Keywords:** Body fat, Energy expenditure, Physical activity, Pregnancy, Metabolic equivalent, Women

## Abstract

**Background:**

Physical activity is associated with health in women. Published MET-values (MET: metabolic equivalent of task) may assess physical activity and energy expenditure but tend to be too low for subjects with a high total body fat (TBF) content and therefore inappropriate for many contemporary women. The MET-value for an activity is the energy expenditure of a subject performing this activity divided by his/her resting energy expenditure, often assumed to be 4.2 kJ/kg/h. Relationships between TBF and MET have been little studied although overweight and obesity is common in women. Available data indicate that MET-values decrease during pregnancy but more studies in pregnant contemporary women are needed.

**Subjects and methods:**

Using indirect calorimetry we measured energy expenditure and assessed MET-values in women, 22 non-pregnant (BMI: 18–34) and 22 in gestational week 32 (non-pregnant BMI: 18–32) when resting, sitting, cycling (30 and 60 watts), walking (3.2 and 5.6 km/h) and running (8 km/h). Relationships between TBF and MET-values were investigated and used to predict modified MET-values. The potential of such values to improve calculations of total energy expenditure of women was investigated.

**Results:**

The resting energy expenditure was below 4.2 kJ/kg/h in both groups of women. Women in gestational week 32 had a higher resting energy metabolism (*p* < 0.001) and 7–15% lower MET-values (*p* < 0.05) than non-pregnant women. MET-values of all activities were correlated with TBF (*p* < 0.05) in non-pregnant women and modified MET-values improved estimates of total energy expenditure in such women. In pregnant women, correlations (*p* ≤ 0.03) between TBF and MET were found for running (8 km/h) and for walking at 5.6 km/h.

**Conclusions:**

Our results are relevant when attempts are made to modify the MET-system in contemporary pregnant and non-pregnant women. MET-values were decreased in gestational week 32, mainly due to an increased resting energy metabolism and studies describing how body composition affects the one MET-value (i.e. the resting energy metabolism in kJ/kg/h) during pregnancy are warranted. Studies of how pregnancy and TBF affect MET-values of high intensity activities are also needed. Corrections based on TBF may have a potential to improve the MET-system in non-pregnant women.

## Background

The physical activity of men and women is extensively studied since it is associated with chronic disease [1]. Of special interest is that physical activity during pregnancy tends to counteract preeclampsia, gestational diabetes and excessive weight gain [2–4]. The positive effect of physical activity is, at least partly, mediated by the increased energy expenditure and this increase may be used to assess how physically active human subjects are. Thus, the metabolic equivalent of task (MET)-system [5–7] is useful if the time spent in different activities is known. A MET-value is then assigned to each activity describing it’s intensity. Such information can, if the resting energy expenditure (the basal metabolic rate, BMR, or the resting metabolic rate) is known, be used to calculate the total energy expenditure (TEE). Estimates of TEE are required when conducting nutritional evaluation and when evaluating the validity of energy intakes [8].

The MET-value of an activity is often defined as the energy expenditure of a subject when performing this activity divided by his/her resting energy expenditure [9]. Published MET-values [5–7] assume the latter to be 4.2 kJ (1 kcal) per kg body weight and hour [10]. However, body composition accounts for a large part of the variation in the resting energy metabolism and published MET-values may be too low for overweight subjects [9]. McMurray et al. [11] reported the average resting metabolic rate of women to be only 0.84 kcal per kg body weight and hour which may lead to errors in the MET-system and Kozy et al. [12] proposed a correction to increase MET-values of overweight subjects. It has been noted [13–15] that published MET-values are too low for subjects with a high body fat content but, to our knowledge, there are no systematic studies on the relationship between total body fat (TBF) and MET-values.

The effect of variations in the body fat content on MET-values is of special concern in women. The prevalence of overweight and obesity in female populations has increased during recent years [16] and the body fat content may be quite variable [17]. Furthermore, the correction proposed by Kozy et al. [12] may not be appropriate since the equation used to predict BMR tends to be inaccurate in females [18]. Finally, insufficient information is available regarding effects of pregnancy and body fat on MET-values.

Studies in pregnant women have sometimes been conducted using MET-values developed in non-pregnant subjects [19, 20]. However, during gestation, both TEE and BMR increase [21]. Prentice et al. [21] compiled studies of how weight bearing and non-weight bearing activities affect energy expenditure during pregnancy and found that MET-values decrease towards the end of pregnancy mainly due to increased BMR. The results of the studies in their review [21] varied considerably, possibly due to variations in design and intensities of the investigated activities. Furthermore, some of these studies were conducted in developing countries and some before the worldwide increase in the prevalence of overweight and obesity. It was recommended that the MET-system should not be used in pregnancy without modification [21] but there is little help available when formulating such modifications. This paper attempts to suggest some guidelines in this area.

We recruited two groups of women, one pregnant and one non-pregnant to a study with the following two objectives: 1) To assess the effect of gestational week 32 on MET-values for activities with varying intensity; 2) To identify relationships between TBF and MET-values for such activities. Using previously published data [22], we had a third objective: 3) To investigate the possibility to improve calculated TEE before pregnancy and in gestational week 32 using relationships between TBF and MET-values, identified in objective 2.

## Methods

### Objectives 1 and 2

#### Subjects and study outline

Forty-four healthy, non-smoking women (22 non-pregnant and 22 pregnant) were recruited during 2007 and 2008 by means of advertisements in the local press. Results obtained in some of these women have been published [23, 24]. All pregnant women had uncomplicated pregnancies and were investigated in gestational week 32. Age, occupation and exercise habits were assessed using a questionnaire. For women in gestational week 32 the questionnaire targeted the situation before pregnancy and included weight before conception. After delivery these women were asked about weight and length at birth of their infant and about their maximal weight during gestation. For women in the pregnant group recorded information were, as far as possible, confirmed using medical records. All women collected two or three urine samples at home and brought those to the measurement session which started with an assessment of BMR, body weight (KCC 150, Mettler-Toledo, Albstadt, Germany) and height. Then women consumed a breakfast containing cornflakes, juice and 3% fat milk providing 30 kJ per kg body weight with 15% energy from protein. After breakfast women performed six standardised activities when their energy expenditure was measured. These activities were: sitting, walking on a treadmill (3.2 and 5.6 km/h), running on a treadmill (8 km/h) and cycling at 30 and 60 watts. The woman could terminate vigorous activities earlier if she felt any discomfort. One pregnant woman did so when walking at 5.6 km/h. One non-pregnant and 13 pregnant women declined the running activity. After this session women received a dose of water, labelled by stable isotopes and were asked to collect six urine samples during the following 14 days carefully noting the time of sampling.

#### Assessment of energy expenditure and MET-values

To assess BMR, oxygen consumption (VO_2_) and carbon dioxide production (VCO_2_) were measured during 20 min in the supine position after an overnight fast and 45 min of rest using a ventilated hood system (Deltratrac Metabolic Monitor, Datex Instrumentarium Corp, Helsinki, Finland). To assess energy expenditure when performing standardised activities a spirometer (CPX/D, Spiropharma, Klampenborg, Denmark) was used to measure VO_2_ and VCO_2_ during 15 s-periods for six minutes. The woman was connected to the spirometer through a mouthpiece and wore a nose clip. The session started with the sitting activity followed by standing. The woman was then transferred to a treadmill board, adjusted to make her walk at 3.2 km/h followed by walking at 5.6 km/h and then running at 8 km/h. Subsequently she was allowed to rest for 20 min after which she mounted a test bicycle where she cycled at 30 watts and then at 60 watts. VO_2_ and VCO_2_ were recorded throughout the six minutes when each standardised activity was performed and steady state was always reached after two to three minutes. The validity of the response, both of the Deltatrac and the Spiropharma, was controlled according to instructions provided by the manufacturer. This involved calibration using gases with defined concentrations of O_2_ and CO_2_ before each test. For each activity average of values recorded during the fifth of the six minutes was used to calculate energy expenditure and MET-values. The recorded VO_2_ and VCO_2_ values confirmed that all subjects were in a steady state when values used to calculate energy expenditure were recorded. Energy expenditure was calculated using the Weir equation [25] and MET-values as energy expenditure of a woman when performing a standardised activity divided by her BMR.

#### Assessment of TBF

Each woman consumed an accurately weighed dose of stable isotopes (0.09 g ^2^H_2_0 and 0.23 g H_2_^18^0 per kg body weight) per os. Isotope enrichment of dose and urine samples was analyzed as previously described [13]. ^2^H-space (N_D_) and ^18^O-space (N_O_) were calculated from zero-time enrichments obtained from the exponential isotope disappearance curves providing estimates of the fractional turnover rates for the two isotopes [13], i.e. the back-extrapolation approach [26]. Analytical precision for results in ppm were 0.22 for ^2^H and 0.03 for ^18^O. Total body water was calculated as (N_D_/1.04 + N_O_/1.01)/2 and fat-free mass as total body water divided by 0.732 (non-pregnant) [26] or 0.747 (gestational week 32) [27]. TBF was body weight minus fat-free mass. N_D_ / N_O_ was 1.032 ± 0.005 (*n* = 44). Analyzing dose and urine samples from one subject nine times gave the following CV: 0.3% (total body water), 0.3% or less (fractional turnover rates) all well within the recommended criteria [28]. Assessment of total body water and calculation of body fat using the two-component model provides valid body composition data in healthy non-pregnant adults [26] and in gestational week 32 [27].

#### Sample size justification

When comparing MET-values of pregnant and non-pregnant women, 20 subjects in each group gave a power above 0.8 to detect a difference of 10% between groups while assuming the CV of MET-values to be 10%. A correlation coefficient of at least 0.45 for linear relationships between MET-values and TBF would be significant with 20 observations.

### Objective 3

We have previously investigated and published the physical activity pattern and TEE of 23 women before pregnancy and in gestational week 32 [22]. Before pregnancy their mean age was 30 ± 4 years, body weight 67.2 ± 12.0 kg, BMI 24.2 ± 4.8 kg/m^2^ and TBF 32.7 ± 7.8%. Their TEE was assessed during 14 days using the doubly labeled water (DLW) method (TEE_DLW_) [22]. A few days after this assessment the women completed a questionnaire recording time spent in the following physical activity categories: sleeping, very light, light, moderate, vigorous and very vigorous [22]. The questionnaire covered all 14 days (20,160 min) when TEE_DLW_ was assessed [22]. In the present paper we use this recorded activity pattern to calculate TEE of the 23 women [22] during the 14 day period using either published MET-values (TEE_pubMET_) or modified MET-values, predicted by means of the relationships between TBF (%) and MET-values (TEE_modMET_) identified in the present study. Then TEE_pubMET_ and TEE_modMET_ are both compared to TEE_DLW_.

### Statistics

Values are given as means ± standard deviations (SD). Significant differences between mean values were identified using *t*-test for paired or unpaired observations after confirming that values were normally distributed. If a normal distribution could not be confirmed Wilcoxon’s test was used. Linear regression analysis was also used. Significance (two-sided) was accepted when *p* < 0.05. Statistical analyses were conducted using Statistica, version 8.0 (Statsoft, Scandinavia AB, Uppsala, Sweden).

## Results

### Objectives 1 and 2

#### Characteristics of women

The pregnant and non-pregnant women recruited to the study are described in Table 1. Means and ranges for age, height and non-pregnant BMI were similar among the two groups. Before conception, pregnant women were engaged in similar occupations and had similar exercise habits as the non-pregnant women. On average pregnant women (*n* = 22) gained 12 ± 4 kg body weight during the complete pregnancy and all of them delivered one healthy full-term infant, weight and length at birth being 3410 ± 390 g and 50 ± 2 cm, respectively.

**Table 1:** Characteristics of non-pregnant and pregnant^a^ women^b^

	Non-pregnant (*n* = 22)		Pregnant^a^ (*n* = 22)	
Age (years)^c^	35 ± 7	(20–45)	32 ± 4	(24–41)
Body weight (kg)^d^	67.2 ± 13.3	(47.1–101.6)	74.2 ± 8.8	(52.4–91.2)
Height (m)^c^	1.69 ± 0.06	(1.55–1.81)	1.67 ± 0.06	(1.59–1.83)
Prepregnancy body weight (kg)	–	–	66 ± 9	(47–85)
Non-pregnant BMI (kg/m^2^)^d^	23.4 ± 4.0^e^	(17.7–33.6)^e^	24 ± 3^f^	(18–32)^f^
Total body fat (%)	29.8 ± 7.7	(12.2–43.8)	32.1 ± 5.4	(19.9–40.7)
Occupation^g^				
Office work	10 (45)		11 (50)	
Nursing	5 (23)		4 (18)	
Child care	4 (18)		4 (18)	
Other	3 (14)		3 (14)	
Regular physical activity^g^				
Never	5 (23)		4 (18)	
1–2 times a week	5 (23)		7 (32)	
> 2 times a week	12 (55)		11 (50)	

^a^ In gestational week 32; ^b^ Values are means ± standard deviations and (ranges) for continuous variables and n (%) for categorical variables; ^c^ Pregnant and non-pregnant women are not significantly different (*p* > 0.05) by independent *t*-test; ^d^ Values of non-pregnant women are not significantly different (*p* > 0.05) from prepregnancy values of pregnant women by independent *t*-test; ^e^ In the non-pregnant group four (18%) of the women were overweight (BMI = 25–29.9) and two (9%) were obese (BMI ≥ 30); ^f^ Based on pre-pregnant values, five women (23%) were overweight (BMI = 25–29.9) and one (5%) was obese (BMI ≥ 30); ^g^ In the pregnant group the figures refer to the situation before pregnancy

#### Energy expenditure at rest and during standardised activities for pregnant and non-pregnant women

Table 2 shows VO_2_, VCO_2_ and energy expenditure at rest (BMR) and when performing the standardised activities. For pregnant women, BMR in kJ/kg/h was 94% of 4.2 kJ/kg/h and the corresponding value for non-pregnant women was 88%. When expressed in kJ/min, BMR was significantly (*p* = 0.000037) and 18% higher of pregnant than of non-pregnant women. For all activities except running energy expenditure (kJ/min) of the pregnant women was slightly higher than the corresponding values for the non-pregnant women but only the values for walking at 5.6 km/h were significantly different. In pregnant women, energy expenditure minus resting energy expenditure (the net energy expenditure) was on average 0.60 ± 0.58, 9.84 ± 0.89, 16.2 ± 1.0, 10.1 ± 1.0, 19.7 ± 2.7 (*n* = 21) and 36.4 ± 4.4 (*n* = 9) kJ/min when sitting, cycling at 30 and 60 watts, walking at 3.2 and 5.6 km/h and running at 8 km/h, respectively. In the non-pregnant group the corresponding values were: 1.04 ± 0.35, 10.1 ± 0.8, 16.2 ± 0.9, 10.1 ± 1.9, 18.2 ± 3.4 and 37.3 ± 5.9 (*n* = 21) kJ/min, respectively. The value for sitting was significantly lower for pregnant women (*p* = 0.0039). No significant differences between the groups were found for any other activity.

**Table 2:** Oxygen consumption, carbon dioxide production and energy expenditure of non-pregnant (*n* = 22) and pregnant^a^ (*n* = 22) women at rest and when performing standardised activities^b^

Activity	Oxygen consumption (ml/min)		*p* ^c^	Carbon dioxide production (ml/min)		*p* ^c^	Energy expenditure (kJ/min)		*p* ^c^
	Non-pregnant	Pregnant^a^		Non-pregnant	Pregnant^a^		Non-pregnant	Pregnant^a^	
Resting	208 ± 25	247 ± 30	0.000029	169 ± 2	192 ± 23	0.00078	4.10 ± 0.50^d^	4.85 ± 0.59^e^	0.000037
Sitting	250 ± 25	264 ± 42	0.184	232 ± 23	249 ± 44	0.120	5.15 ± 0.50	5.44 ± 0.88	0.156
Cycling									
30 watts	694 ± 51	717 ± 56	0.157	620 ± 60	660 ± 48	0.020	14.1 ± 1.1	14.7 ± 1.1	0.096
60 watts	980 ± 62	1007 ± 65	0.177	936 ± 63	995 ± 81	0.010	20.3 ± 1.3	21.0 ± 1.4	0.082
Walking									
3.2 km/h	699 ± 110	731 ± 105	0.332	614 ± 107	636 ± 93	0.470	14.2 ± 2.3	14.9 ± 2.1	0.355
5.6 km/h	1082 ± 179	1194±139^f^	0.028	1012 ± 193	1122±167^f^	0.054	22.3 ± 3.8	24.6 ± 3.0^f^	0.032
Running									
8 km/h	1958±290^f^	1948±200^g^	0.931	2045±359^f^	2041±301^g^	0.980	41.3 ± 6.2^f^	41.2 ± 4.6^g^	0.943

^a^In gestational week 32

^b^Values are means ± standard deviations

^c^*p* for difference between groups using unpaired *t*-test

^d^Representing the basal metabolic rate and equivalent to 3.12 ± 0.30 ml O_2_/kg/min and 3.71 ± 0.33 kJ/kg/h

^e^Representing the basal metabolic rate and equivalent to 3.34 ± 0.25 ml O_2_/kg/min and 3.94 ± 0.30 kJ/kg/h

^f^*n* = 21

^g^*n* = 9

#### MET-values of pregnant and non-pregnant women

Table 3 shows MET-values for the standardised activities assessed in the two groups of women. For the walking and running activities our values for non-pregnant women tended to be slightly higher than published values. All MET-values were significantly lower for pregnant than for non-pregnant women. For sitting, and for cycling at 30 and 60 watts, values for pregnant women were 11, 12 and 12% lower than the corresponding values for non-pregnant women, respectively. Lower figures were also obtained for walking at 3.2 km/h (11%), at 5.6 km/h (7%) and for running (15%).

**Table 3:** MET-values^a^ obtained when six standardised activities were performed by non-pregnant women and by pregnant women in gestational week 32^b^

Activity	MET-values^a^		*p*-value^c^	Published MET-value^d^
	Non-pregnant (*n* = 22)	Pregnant (*n* = 22)		
Sitting	1.26 ± 0.10	1.12 ± 0.12	0.00017	1.3
Cycling				
30 watts	3.48 ± 0.24	3.06 ± 0.29	0.000007	3.5^e^
60 watts	4.99 ± 0.43	4.37 ± 0.47	0.00004	4.8^f^
Walking				
3.2 km/h	3.47 ± 0.32	3.07 ± 0.34	0.0003	2.5
5.6 km/h	5.43 ± 0.54	5.04 ± 0.5^g^	0.018	4.3
Running 8 km/h	10.20 ± 0.94^g^	8.67 ± 0.89^h^	0.00026	8.3

MET, metabolic equivalent of task; ^a^ Energy expenditure of a woman when performing a specific activity divided by her basal metabolic rate as described in subjects and methods; ^b^ Values are means ± standard deviations; ^c^ *p* for difference between pregnant and non-pregnant groups using unpaired *t*-test; ^d^ For non-pregnant individuals [7];

^e^ Cycling at 30–50 watts; ^f^ Cycling at 51–89 watts; ^g^ (*n* = 21); ^h^ (*n* = 9)

#### Relationships between TBF and MET-values

Table 4 shows that, for non-pregnant women, significant linear relationships for all standardised activities were found when measured MET-values were regressed on TBF (%). For sitting and for cycling at 30 and 60 watts, these relationships were negative (r ranging from − 0.44 to − 0.59) while for walking, both at 3.2 and 5.6 km/h, and for running they were positive (r ranging from 0.53 to 0.56). Significant correlations were also found for non-pregnant women when measured MET-values were regressed on TBF (kg), with r ranging from − 0.61 to − 0.68 (*p* ≤ 0.003) for sitting and cycling activities and from 0.43 to 0.52 (*p* ≤ 0.044) for walking and running activities. For pregnant women significant correlations between MET and TBF (%) were found for the running activity (*n* = 9) (*r* = 0.78, *p* = 0.012) and for walking at 5.6 km/h (*n* = 21) (*r* = 0.47, *p* = 0.03). The correlation between MET and TBF (kg) was significant for the running activity (*n* = 9) (*r* = 0.82, *p* = 0.007). No other significant correlations between MET and TBF (%, kg) were found in pregnant women.

**Table 4:** MET-values^a^ (y) regressed on total body fat (%) (x) for non-pregnant women (*n* = 22) when performing standardised activities^b^

Equation no	Activity	Slope	Intercept	*r*	*p*
1.	Sitting	− 0.0077	1.489	−0.59	0.004
2.	Cycling, 30 watts	−0.0162	3.963	−0.52	0.014
3.	Cycling, 60 watts	−0.0246	5.725	−0.44	0.042
4.	Walking, 3.2 km/h	0.0225	2.795	0.54	0.009
5.	Walking, 5.6 km/h	0.0393	4.261	0.56	0.006
6.	Running, 8 km/h^c^	0.0682	8.222	0.53	0.015

*MET* Metabolic equivalent of task; ^a^ Energy expenditure of a woman when performing a specific activity divided by her basal metabolic rate as described in subjects and methods; ^b^ Values given are slopes and intercepts of the regression line and correlation coefficients (*r*) with their *p*-values; ^c^ (*n* = 21)

### Objective 3

#### Investigating the possibility to improve calculated TEE

All investigated correlations between measured MET-values and TBF were significant for non-pregnant women while only a few such correlations were significant for pregnant women. Therefore, we investigated the possibility to improve calculations of TEE only in non-pregnant women. As shown in Table 5, we used regression equations in Table 4 to predict modified MET-values for each activity category. These values as well as published MET-values were then used to calculate energy expenditure of the women in our previous study [22] when they were in the pre-pregnant state. For the very light activity category we used eq. 1 in Table 4, a calculation which produced significantly lower energy expenditure than did the corresponding calculation based on the published MET-value. As also shown in Table 5, we used eqs. 4, 5 and 6 in Table 4 for moderate, vigorous and very vigorous activity categories, respectively. We selected these equations based on knowledge of the physical activity pattern of the women in our previous study [22] where the predominant activities were walking and running rather than cycling. Table 5 shows that for each of these three activity categories women were found to expend significantly more energy when the modified rather than the published MET-values were used. Finally, as also shown in Table 5, when energy expenditure during 24 h was calculated TEE_pubMET_ was 9710 ± 2110 kJ/24 h and significantly lower than TEE_DLW_. This value was 10,510 ± 1500 kJ/24 h [22] while TEE_modMET_ was 10,060 ± 2660 kJ/24 h and not significantly different from TEE_DLW_.

**Table 5:** Minutes per 24 h and energy expenditure in six activity categories^a^ calculated using published [7] and modified^b^ MET-values^c^

Activity category	Minutes per 24 h	Published MET-values	Modified MET-values	Energy expenditure calculated using published MET-values (kJ)^d^	Energy expenditure calculated using modified MET-values (kJ)^d^
Sleeping	458 ± 61	0.9	0.9	1557 ± 299	1557 ± 299
Very light	446 ± 250	1.4	1.23 ± 0.06^e^	2285 ± 1216	2022 ± 1076^f^
Light	300 ± 219	2.4	2.4	2803 ± 2264	2803 ± 2264
Moderate	187 ± 178	3	3.53 ± 0.18^g^	2140 ± 2080	2535 ± 2480^h^
Vigorous	41 ± 47	4.5	5.54 ± 0.3^i^	690 ± 790	849 ± 979^h^
Very vigorous	8 ± 14	8	10.4 ± 0.53^j^	232 ± 451	292 ± 563^k^
Total per 24 h	1440			9710 ± 2110^l^	10,060 ± 2660^m^

*BMR,* Basal metabolic rate, *TEE* Total energy expenditure, *MET* Metabolic equivalent of task

^a^ Based on information in a questionnaire recording the physical activity pattern during 14 days of 23 non-pregnant healthy Swedish women as described in subjects and methods and in [22]; ^b^ Each women was given a modified MET-value which represented a MET-value predicted from her body fat content (%) using the appropriate equation in Table 4 except for sleeping and the light activity categories where the modified MET-values were equal to the published MET-values, i.e. 0.9 (sleeping) and 2.4 (light activity) for all women; ^c^ Values are means ± standard deviations; ^d^ Calculated using the appropriate MET-value multiplied by the BMR of the women during the time period when the particular activity was performed; ^e^ Obtained using eq. 1 in Table 4; ^f^ Significantly lower (*p* < 0.05) than the corresponding value obtained using published MET-values, assessed using paired *t*-test; ^g^ Obtained using eq. 4 in Table 4; ^h^ Significantly higher (*p* < 0.05) than the corresponding value obtained using published MET-values, assessed using paired *t*-test; ^i^ Obtained using eq. 5 in Table 4; ^j^ Obtained using eq. 6 in Table 4; ^k^ Significantly higher (*p* < 0.05) than the corresponding value obtained using published MET-values, assessed using a paired non-parametric test (Wilcoxon); ^l^ 800 ± 1800 kJ/24 h lower (*p* < 0.05) than TEE, estimated using doubly labelled water [22], assessed using paired *t*-test; ^m^ 450 ± 2270 kJ/24 h lower (*p* > 0.05) than TEE, estimated using doubly labelled water [22], assessed using paired *t*-test

## Discussion

### Summary of main results

In healthy women we found MET-values to be lower in gestational week 32 than in non-pregnant women, an observation mainly explained by the increased BMR during pregnancy. We identified significant relationships between TBF and measured MET in non-pregnant women while for pregnant women such relationships were found for activities with high intensity but not for activities of lower intensity. When relationships between measured MET and TBF were used to predict modified MET-values calculated TEE of our non-pregnant women was improved.

### Comments on design and methods

When the inferences of our results are considered body fatness of our women becomes of interest. The average as well as the variability in their body fat content represents relevant information. In population studies body fatness is commonly assessed as BMI rather than by means of a suitable body composition method. This may be appropriate since relationships between BMI and TBF (%) have been identified [17] but is not always satisfactory since this relationship may be different in different populations [29]. In our study overweight and obesity was present in 27 and 28% of non-pregnant and pregnant women, respectively. This is slightly lower than such figures for contemporary Swedish women [30] but higher than this value in most of the studies of MET-values during pregnancy as reviewed by Prentice et al. [21]. Furthermore, the variability in TBF (%) expressed as CV, was 25.8 and 16.8% for our non-pregnant and pregnant women, respectively. This is similar to corresponding previously reported values, 23.5 [13] and 16.9 [31] % for non-pregnant women and for women in gestational week 32, respectively. These comparisons indicate that the body composition of our women may be quite typical for many contemporary women. However, our samples are small and recruited from only one area in Sweden. It should also be emphasized that contemporary populations of women may well have a lower, as well as a higher, body fatness. Unfortunately, there is a lack of body composition studies in such populations making this kind of comparisons difficult.

When assessing activity energy expenditure of a woman to establish MET-values it is relevant to consider her feeding status. In the fed state the recorded energy expenditure will include dietary induced thermogenesis i.e. the increase in energy expenditure following food intake which represents about 10% of the energy content of the food consumed and lasts for at least six hours [32]. Published MET-values are often assessed in the fed state [5, 33] and therefore include dietary induced thermogenesis. Our women were fed a standardised breakfast and thus MET-values of both groups would be equally affected by dietary induced thermogenesis.

The four-component model represents the best available method to assess body composition in vivo of human subjects while two- and three-component models are less accurate because they rely on assumptions [34]. Assessment of total body water and application of the two-component model to calculate fat-free mass and subsequently TBF assumes that the degree of hydration is known and relatively constant, assumptions generally considered acceptable for healthy adults [26]. Löf and Forsum investigated these assumptions in pregnant women and concluded that this way to assess body composition is as least as valid in gestational week 32 as it is in non-pregnant subjects [27]. The validity of these assumptions during other parts of pregnancy has not been satisfactorily confirmed. Therefore we selected gestational week 32 of pregnancy for our study. This assessment of TBF will include the TBF of the fetus. However, in gestational week 32, this represents only about 0.4% of the TBF of the mother [31].

This study is focused on the relationship between TBF and MET-values since published research shows that body fatness is a relevant factor when MET-values are assessed and applied. Note, however, that our study does not provide a complete description of how body composition and MET-values interact.

### Effects of pregnancy in gestational week 32 on MET-values

For all investigated non-resting activities differences in energy expenditure between pregnant and non-pregnant women were small and the net increase in energy expenditure tended to be quite similar for the two groups. However, all measured MET-values were significantly lower in gestational week 32. Obviously, this is due to the increased BMR at this stage of pregnancy.

An effect of pregnancy is to increase body weight and it has been suggested that pregnancy influences MET-values of weight bearing and non-weight bearing activities differently. For a non-weight bearing activity such as cycling, we found MET-values to be on average 0.42 (30 watts) and 0.62 (60 watts) units lower for pregnant than for non-pregnant women. This effect is similar to the corresponding average effect in gestational week 32 obtained in the studies compiled by Prentice et al. [21]. For weight bearing activities, such as walking (both at 3.2 and 5.6 km/h) our average MET-values were 0.4 units lower for pregnant than for non-pregnant women. Again this agrees reasonably well with the estimated effect of pregnancy in gestational week 32 on MET-values for walking [21]. However, we also observed that the measured MET-value for a vigorous activity, i.e. running at 8 km/h, was as much as 1.5 units lower for pregnant than for non-pregnant women suggesting that the effect of pregnancy on MET-values is influenced by the intensity of the activity. Thus our findings for women in gestational week 32 confirm and extend previously presented observations [21].

### Relationships between MET and TBF

All relationships between MET-values and TBF were significant in non-pregnant women, while only relationships for activities with higher intensity were significant for pregnant women. This may reflect that the influence on energy metabolism by factors associated with pregnancy, such as fetal metabolism, overrides the influence of TBF unless the activity is of sufficient intensity.

In our study, sitting and cycling can be considered to represent non-weight bearing activities while walking and running represent weight bearing activities. For non-pregnant women it is of interest to note that relationships between MET and TBF (%) were negative for sitting activities (including cycling) and positive for walking and running. This suggests that TBF may affect MET-values for the two kinds of activities differently but studies with a larger selection of activities are required for firm conclusions on this issue.

### Application of the MET-system during pregnancy

This study confirmed that the increase in BMR is an important factor behind the effect of pregnancy on MET-values. Thus it becomes important to investigate how pregnancy influences the one MET-value. Using published data [35] we found BMR to be equivalent to 81% of the conventional MET-value before pregnancy and until gestational week 20. This value increased to 87 and 88% in gestational weeks 32 and 35, respectively. In the present study, this figure was 94% in gestational week 32. Melzer et al. [36] showed this figure to be close to 100% in gestational weeks 35–41. This value is also likely to differ between populations which, for various reasons, differ in body fatness. There is a lack of studies of how the one MET-value varies during the course of pregnancy in populations with variations in body composition.

Our observations suggest that the effect of pregnancy on MET-values may be influenced by the intensity of the activity and also that body composition may influence MET values of high intensity activities. Consequently, there is a need for systematic studies regarding the effect of body fatness and activity intensity on MET-values during pregnancy if appropriate modifications of the MET-system for pregnant women are to be developed.

### BMR, MET and calculated TEE of non-pregnant women

Women as well as overweight and obese subjects tend to have BMR below 4.2 kJ/kg/h [11] and high MET-values when these are assessed using measured resting energy expenditure. The BMR of our non-pregnant women was also low and their MET-values tended to be high (Table 3).

This study showed that modified MET-values, predicted using the relationships in Table 4, improved calculated TEE in non-pregnant women. However, depending on the activity category, energy expenditure calculated using modified MET-values were either lower (very light activity) or higher (moderate, vigorous and very vigorous activity) than energy expenditure calculated using published MET-values. According to Table 4, TBF (%) is negatively associated with MET-values in eq. 1, which is used for very light activity, but positively associated with MET-values in eqs. 4, 5 and 6 which are used for moderate, vigorous and very vigorous activities, respectively. Our results suggest that a correction based on the TBF content may represent a possible way to improve the MET-system for women. More work is needed to find out if such a correction is superior to the correction proposed by Kozy et al. [12] which is based on BMI and resting energy metabolism predicted using weight, height and age.

### **Strengths a**n**d limitations of this study**

Strengths are that we measured MET-values of non-pregnant and pregnant women, both with valid estimates of TBF, and that both non-weight bearing and weight bearing activities were included. In non-pregnant women we established relationships between TBF (%) and measured MET-values for standardised activities and demonstrated that such relationships may improve calculated TEE. A limitation of our study is the small number of subjects.

## Conclusion

A valid MET-system is important when physical activity levels and energy intake of human subjects are assessed. This kind of information is needed by health workers and researchers within several different areas, for example nutrition and public health. This study deals with issues related to application of the MET-system and provides suggestions for improving this system. Correcting MET-values based on TBF of subjects may represent a possibility to improve this system for non-pregnant women. Our results indicate that for pregnant women more work is needed to develop a valid MET-system. Studies describing how body composition influences the one MET-value throughout gestation and how pregnancy and body fat affect MET-values for high intensity activities may be useful in this context.

## Abbreviations

BMR: Basal metabolic rate
DLW: Doubly labelled water
MET: Metabolic equivalent of task
N_D_: ^2^H dilution space
N_o_: ^18^O dilution space
TBF: Total body fat
TEE: Total energy expenditure
TEE_DLW_: Total energy expenditure assessed using doubly labelled water
TEE_modMET_: Total energy expenditure calculated using modified MET-values
TEE_pubMET_: Total energy expenditure calculated using published MET-values
VCO_2_: Carbon dioxide production
VO_2_: Oxygen consumption
